# Dual-Layer Approach toward Self-Healing and Self-Cleaning Polyurethane Thermosets

**DOI:** 10.3390/polym11111849

**Published:** 2019-11-09

**Authors:** Muhammad Naveed, Muhammad Rabnawaz, Ajmir Khan, Mohammad O. Tuhin

**Affiliations:** School of Packaging, Michigan State University, 448 Wilson Road, East Lansing, MI 48824-1223, USA; naveedmu@msu.edu (M.N.); khanajmi@msu.edu (A.K.); tuhinmo1@msu.edu (M.O.T.)

**Keywords:** polyurethanes, propyl gallate, sustainable materials, self-healing, self-cleaning surfaces, durability

## Abstract

There is an urgent need for coatings that exhibit both self-healing as well as self-cleaning properties as they can be used for a wide range of applications. Herein we report a novel approach toward fabricating polyurethane thermosets possessing both self-cleaning and self-healing properties. The desired coating was achieved via casting a bottom layer of self-healable polyurethanes comprised of reversible phenolic urethane bonds followed by a subsequent dip-coating of the prepared layer in a solution of bis(3-aminopropyl)-terminated polydimethylsiloxane (PDMS-NH_2_). The PDMS was used to impart self-cleaning properties to the coating. While the self-healing behavior of the bottom polyurethane layer is achieved through phenolic urethane chemistry, via the exchange of phenolic urethane moieties. The prepared coatings were tested for their optical, mechanical, self-healing, and self-cleaning properties using a variety of characterization methods, which confirmed the successful fabrication of novel self-cleaning and self-healing clear urethane coatings.

## 1. Introduction

Polymeric materials are prone to surface damage during their life cycle. If this damage is left unaddressed, it can propagate with the passage of time and thus can cause a loss of structural integrity, environmental degradation of the substrate, as well as functional and mechanical failure. These types of failures not only shorten the life cycle of that material but also increase the disposal and maintenance costs. A promising approach to reduce cost, waste, and mitigate damage is the use of materials that can undergo self-repair in an analogous manner to that of living organisms. Extensive research has been undertaken to develop effective and inexpensive methods of fabricating self-healing materials that are broadly classified into extrinsic and intrinsic self-healing approaches [1] The former approach involves the encapsulation of healing agents that are released in response to damage or crack formation. Consequently, the cracks are filled by the self-healing agent and hardened by ambient crosslinking. This approach was initially reported by White et al. [2] when they developed a self-healing epoxy matrix by embedding capsules that were filled with a healing agent. In more recent modifications of this strategy, the healing agents can also be incorporated into hollow cylinders and fibers [3], or capsules prepared with microfluidic devices [4]. Although the extrinsic approach has the advantage of offering autonomous self-healing without the need for external assistance, unfortunately, this approach has some disadvantages, such as the exhaustion of the healing agent, instability of the healing agent, and the one-time healing at the damage site. In addition, poor optical clarity owing to light scattering by embedded microcapsules is another drawback of this method.

On the other hand, the intrinsic approach involves the healing of cracks and damage via the rearrangement of the bonds in the materials at the damaged site. Therefore, materials with reversible (dynamic) covalent bonds are employed to impart self-healing properties by this intrinsic approach. A key advantage of the intrinsic approach over extrinsic self-healing is the repeatable healability at the same damaged site. Some well-explored system with dynamic covalent bonds that are often used for self-healing applications include Diels-Alder reaction [5,6], acylhydrazone [7,8,9], transesterification [10,11], disulfide bonds [12,13,14,15], and nitroxide moieties [16]. Other dynamic interactions have also been found to exhibit self-healing behavior, such as host-guest interactions [17,18,19], π–π stacking interactions [20,21,22], and dynamic Hydrogen-bonds [23]

On the other hand, self-cleaning materials - surfaces that repel polar and non-polar liquids have great potential as sensors and for other applications [24,25]. Over the last three decades, extensive research has been devoted toward the fabrication of omniphobic surfaces, and this work has often been inspired by natural phenomena such as lotus leaves [26], water strider legs [27], and pitcher plants [25]. Broadly, self-cleaning approaches can be classified into two categories. The first (and the most widely explored) group are roughly textured self-cleaning surfaces, but these rough surfaces are often plagued with poor durability [28], limited optical clarity [29,30], and they are prone to failure under pressure. The second category deals with smooth self-cleaning surfaces [25,31,32,33,34], which include slippery liquid-infused porous surfaces (SLIPS) [25,35] and non-porous thermosets bearing liquid-like low surface energy components on their surface [36,37]. One of the key drawbacks of the self-cleaning surfaces is the use of fluorochemicals regardless of whether they are smooth- or roughly textured systems. However, due to the harmful and persistent nature of fluorine-containing compounds in the environment, fluorine-free alternatives are highly desirable. In this regard, PDMS has recently been employed by our group and others to introduce self-cleaning properties [38,39,40,41,42,43,44,45]. In a recent study, polydopamine (PDA) coated fabric was treated with stearic acid to impart excellent water repellency. However, these coatings were non-repellent for oil as evident from their use in water and oil separation [46] Also, fluorine-free rough surfaces were recently reported with very high contact angles for water, but these surfaces are neither oil-repellent nor self-healing [47]. As discussed above, both self-cleaning and self-healing materials have been widely explored, but the integration of self-healing and self-cleaning properties into a single material has received little attention.

Although there is a great need for coatings that combine self-healing with self-cleaning properties as they can be used for a wide range of applications, neither academia nor industry has developed a practical solution to this challenge. Herein we report a novel approach toward polyurethane coatings that offer self-healing as well as self-cleaning properties via a dual-layer approach. The desired coating was achieved by first casting a base layer of self-healable polyurethanes comprised of reversible phenolic urethane bonds and the subsequent application of a second layer via dip-coating from PDMS-NH_2_ solution. PDMS was used to impart self-cleaning properties, while the dynamic phenolic urethane chemistry was employed to achieve self-healing properties. The prepared coatings were tested for their optical, mechanical, self-healing, and self-cleaning properties. 

## 2. Experimental Section

### 2.1. Materials

An NCO-terminated poly(tetrahydrofuran) (PTHF)-based polyurethane (PU) pre-prepolymer (with an NCO content of 9.19%), propyl gallate (PG, Sigma Aldrich, purity ≥98%, St Louis, MO, USA), and bis(3-aminopropyl)-terminated polydimethylsiloxane (PDMS, *M*_n_ = 2,500 g/mol, Sigma Aldrich), were used without purification. Similarly, hexamethylene diisocyanate trimer (HDIT, NCO ~17%) was characterized via ^1^H NMR spectroscopy prior to use.

### 2.2. Preparation of the Self-Healing Self-Cleaning Polyurethane Coatings

To prepare the self-healable self-cleaning polyurethane (PU) coatings, PU pre-prepolymer (2.0 g) was first dissolved in US3 (US3 is a mixture of esters and ketones available from Sherwin-Williams, 8.0 mL) in a 20 mL vial. To the above solution, corresponding amounts of propyl gallate (PG) and HDIT (see Table 1) were also added and the reaction mixture was stirred at 100 °C for 40 min. Subsequently, 1.0 mL of this solution was cast onto a glass slide (2.54 × 7.62 cm^2^). The coated glass slide was kept at room temperature until all of the visible solvents had evaporated, and the coating became non-sticky but soft enough to be compressed with a finger. Subsequently, the coated glass was dipped into a PDMS-NH_2_ solution (5.0 mg of PDMS-NH_2_ per mL of hexane) for various durations (e.g., 10, 20, 30, 60 and 120 s).

### 2.3. Characterization

#### 2.3.1. Attenuated Total Reflection-Fourier-Transform Infrared Analysis

ATR-FTIR spectra of the coatings were recorded using a Shimadzu IR Prestige21 FTIR spectrophotometer (Shimadzu Co., Columbia, Maryland, USA) before and after they had been immersed into the PDMS solution. The spectra were recorded in the range of 4000-400 cm^−1^, and a total of 64 scans were recorded for each sample.

#### 2.3.2. X-ray Photoelectron Spectroscopy (XPS)

A PHI 5400 ESCA system equipped with a non-monochromatic mg source for X-ray (take-off angle of 45 degrees, Physical electronics, Inc. Minneapolis, Minnesota, USA) was used to record XPS of the selected samples. Two types of scans were conducted for each sample, including a survey scan in the range of 0–1100 eV, and regional scans for each element identified in the survey scan. The spectra were processed using PHI Multipak (v8.0) software.

#### 2.3.3. Validation of the Self-Healing Behavior

The self-healing capabilities of the samples were monitored by incurring cuts with a razor blade and subsequently observing their healing behavior with an optical microscope (Olympus BX 60 with an Olympus Infinity 2 camera and a 5x objective lens, Olympus optical Corporation, Tokoyo, Japan). The self-healing evaluations were performed at 130 °C for a given period of time.

#### 2.3.4. Water Contact Angle and Hysteresis

Water contact angles (WCAs) were determined using a 590-U1 Goniometer equipped with DROPimage software (Ramé-Hart Instrument Co., Succasunna, New Jersey, USA). Deionized water droplets (10 μL) were placed onto the sample surface and then recorded. The contact angle was measured at three different places on the surface of each sample and the average values were reported. The water contact angle hysteresis values were measured based on the difference between the receding and advancing angle according to Equation (1). The advancing and receding contact angles were recorded via an extension/contraction method by respectively adding and pulling liquid with a needle on the surface of prepared coatings while dynamically measuring the contact angles at both stages.

*Contact Angle Hysteresis* = CosØ*_R_* – CosØ*_A_* (1)

#### 2.3.5. Sliding Contact Angles

A home-built sliding angle measurement instrument was used to determine the sliding angles by placing 80 μL droplets of deionized water on the coating. The surface was gradually tilted until the droplet began to slide. The sliding angles were recorded at three different spots on each sample and average values are reported in this article. Similarly, vegetable oil and hexadecane sliding angles were recorded by using 10 µL droplets of each liquid. As was the case with the waters, these experiments were performed in triplicate and the average value was recorded.

#### 2.3.6. Optical Transmittance

The optical transmittance of the samples prepared in this study was determined with a Perkin Elmer Lambda 25 UV-Vis spectrometer (Shimadzu Corporation, Kyoto, Japan), in which an uncoated glass slide was used as a reference cell. The reported %T values correspond to the wavelength of 540 nm.

#### 2.3.7. Abrasion Tests

The robustness of the self-cleaning performance was studied via measuring contact angle measurements that were performed before and after an abrasion test. The surfaces were rubbed with sandpaper at a loading of 0.343N for ten cycles.

## 3. Results and Discussion

**Figure 1:**
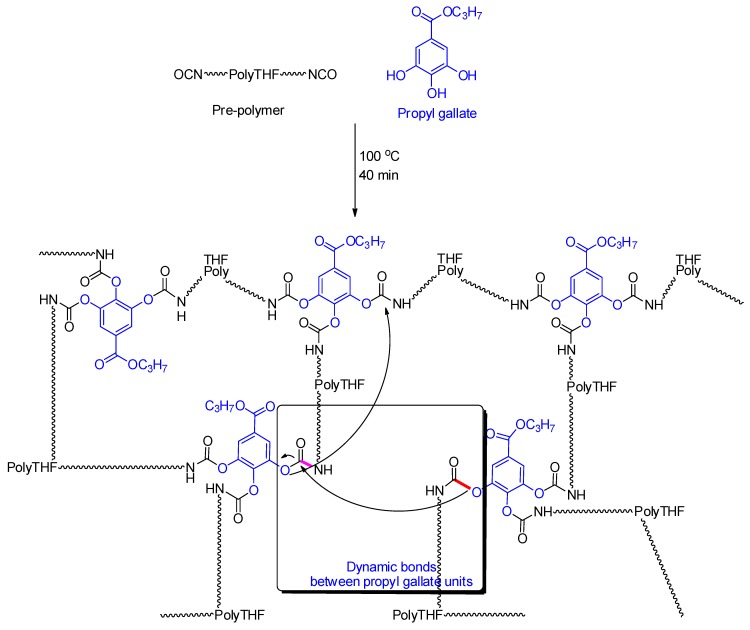
The chemistry of the self-healing PU (bottom layer) prior to modification with PDMS.

The motivation behind this study and choosing urethane was twofold. First, biobased and low-cost propyl gallate was selected as a self-healing agent from the sustainability perspective. Second, the combination of self-healing and self-cleaning properties into single urethane coatings without compromising the desirable optically clarity. The chemistry of the self-healable PU derived from a PU-prepolymer and propyl gallate (PG) is shown in Figure 1. The formation of the urethane bonds derived from the reaction between the phenol moiety of PG and the isocyanate (NCO) group of the PU-prepolymer was reversible at elevated temperatures, and hence these bonds were used to impart healing properties [48]. The arrows in Figure 1 shows the proposed associative-dissociative mechanism for the reversible urethane bonding formed between the phenolic OH and NCO. For the PU coating with reversible phenolic urethane bonds, PG was used to provide the phenolic groups. Further benefits of PG include its low cost and its renewable nature. The PU-prepolymer and PG were characterized via ^1^H NMR spectroscopy as shown in Figures S1 and S2, respectively. Other materials such as HDIT (see Figure S3) and PDMS-2.5K (see Figure S4) were also characterized via ^1^H NMR spectroscopy prior to use.

**Figure 2:**
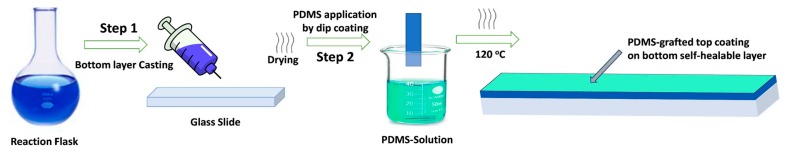
Illustration of the dual-layered approach for preparing a PU coating possessing both self-healing and self-cleaning properties.

To fabricate surfaces exhibiting both self-healing and self-cleaning properties, we used a dual-layer approach as shown in Figure 2. The dual-layer approach was used because we wanted the self-cleaning PDMS polymer to be positioned at the surface or upper layer of this coating, where it can most effectively provide the desired anti-smudge properties. To ensure chemical grafting of the PDMS to the PU matrix, PDMS-NH_2_ was employed as the NH_2_ group reacts with the NCO moieties (of the PU bottom layer) in a quantitative manner. According to our preliminary examinations, a longer period during which the PU coating is immersed into a PDMS solution resulted in the dissolution of the bottom layer. In addition, the use of aggressive solvents for PDMS-NH_2_ (e.g., PDMS-NH_2_ dissolved in acetone) also resulted in the partial dissolution of the PU coating. The initial screening revealed that the optimum results were obtained with the use of hexane as the medium for PDMS-NH_2_ and with an immersion time of 10 s.

**Figure 3:**
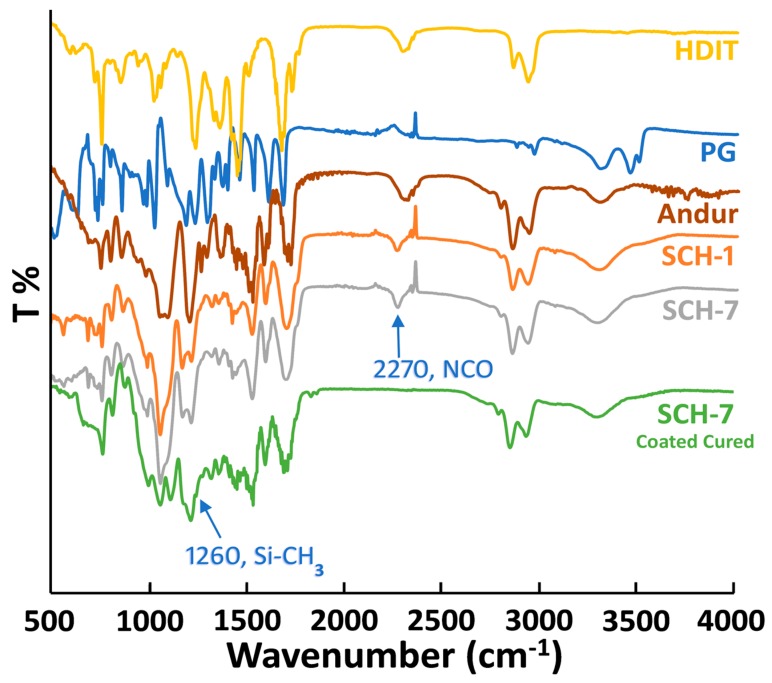
FTIR spectra of the monomers and prepared self-healable bottom layer before and after the grafting of the omniphobic PDMS top layer. Y-axis represents the percentage of transmittance (T %) as a function of wavenumber on the x-axis.

All of the chemical transformations were monitored via ATR-FTIR spectroscopy (see Figure 3). The IR spectra of the PU-prepolymer and HIDT show a characteristic peak at 2270 cm^−1^ corresponding to the NCO group. Similarly, the partially crosslinked PU coating (the bottom layer) was also subjected to IR analysis to confirm the presence of NCO groups, a portion of which was subsequently used for the grafting of PDMS-NH_2_. After the PU bottom layer had been immersed into the PDMS-NH_2_ solution, subsequent curing treatment yielded the finished coating. The IR spectra of this finished coating did not exhibit any NCO peaks at 2270 cm^−1^, indicating a complete curing treatment (SCH-7). In addition, a peak appeared at 1260 cm^−1^ corresponding to the Si-CH_3_ stretching vibration of the indicates the successful grafting of PDMS-NH_2_ [40,49].

**Figure 4:**
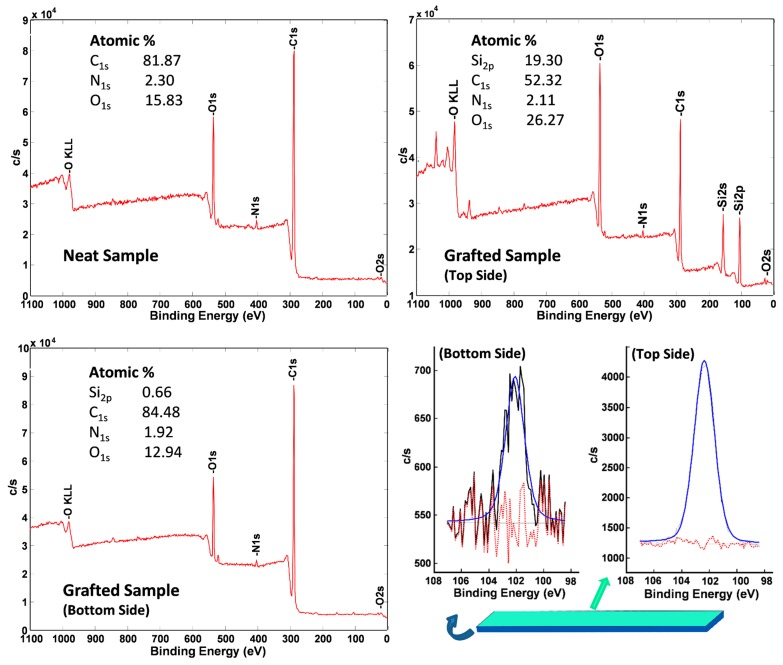
XPS analysis of the PU coatings before (neat) and after PDMS grafting. A self-healable bottom layer was immersed into a solution of PDMS-NH_2_ in hexanes (Concentration: 5.0 mg/1.0mL of solvent) for 10 s. The cured samples were then analyzed via XPS.

In addition to ATR-FTIR studies, the samples were further analyzed via X-ray photoelectron spectroscopy (XPS) to examine the chemical compositions of various elements (see Figure 4). Prior to the XPS analysis, the samples were prepared on a clean high-density polyethylene (HDPE) sheet to avoid contamination by the Si of the glass. As a control, a neat PU coating (sample SCH-4 in Table 1) was examined by XPS both on the front and bottom sides. The spectrum shows the presence of carbon, oxygen, and nitrogen, which are elements present in PU (which is used to prepare the bottom layer). In comparison, the PDMS-treated sample (the front face), showed a strong characteristic Si_2p_ peak at a binding energy of 102 eV with an atomic percentage of 19.30%. The presence of Si peak and the increase in the atomic percentage of oxygen atoms is consistent with the successful grafting of PDMS onto the self-healable PU bottom layer. To confirm whether any diffusion of the PDMS-NH_2_ in the bulk had occurred, the bottom side of the PDMS-treated PU was also characterized via XPS analysis. The bottom side was found to possess a silicon content of only 0.66%. This weak presence of Si indicates that there might be no diffusion of the PDMS down to the bottom face, and the tiny amount of Si may correspond to some impurity peak. Further analysis will be conducted in the future to map the PDMS concentration gradient with respect to distance from the upper surface.

**Figure 5:**
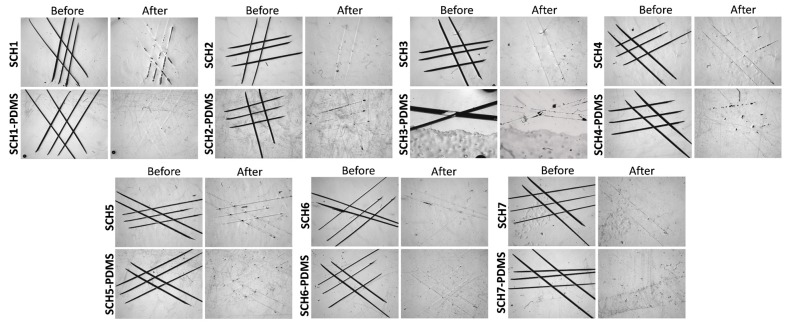
Optical microscopy images showing the self-healing capabilities of various PU with and without PDMS coatings both before and after thermal treatment at 130 °C for 40 min.

Conventional polyurethane cannot undergo self-healing once it has been damaged because it lacks reversible urethane bonds. To demonstrate the reversibility of our PU coatings, the coatings were incurred with cuts by using a razor blade and the damaged surface was allowed to heal at 130 °C for 40 min. Images of the damaged surfaces were taken before and after the healing process with an optical microscope (see Figure 5). It can be clearly seen that the coatings demonstrated strong self-healing capabilities regardless of the presence or absence of PDMS, which suggests that the grafting of PDMS did not affect the self-healing performance of the PU coatings. Despite varying the NCO:OH ratio in the range of 1.0:1.1 to 1.0:0.8, we did not observe any significant differences in the self-healing properties of these materials. This suggests that under the given self-healing conditions, the PU has sufficient urethane dynamicity to enable healing.

To validate the unique self-healing behavior of our prepared PU system, we examined the self-healing ability of conventional non-healing polyurethane coatings. Two non-healable urethane coatings were prepared in this study. The first one was based on PU coating from prepared from the mixing of hexamethylene diisocyanate trimer and acrylic polyol. The second PU system was prepared from poly(tetrahydrofuran) prepolymer terminated with isocyanates groups and glycerol. The second system, in particular, has the same ingredient as used in the self-healing coating reported in this study except that instead of propyl gallate, glycerol was used as polyol. The above two PU coatings did not show any self-healing abilities after thermal treatment at 130 °C for 40 min as depicted in Figures S6 and S7.

**Figure 6:**
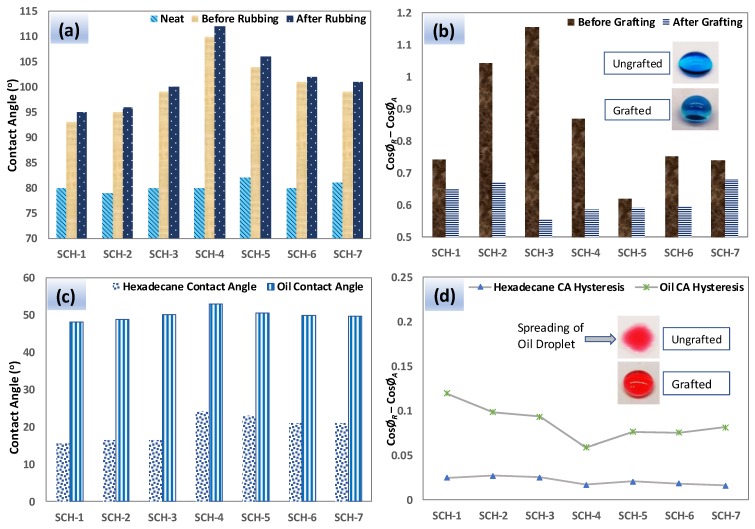
Water contact angles (**a**) and water contact angle hysteresis values (**b**) on various PU surfaces. Also shown are hexadecane and oil contact angle (**c**) as well as Hexadecane and oil contact angle hysteresis values (**d**) on various PDMS-treated PU coatings. Hexane and vegetable oil readily spread on the neat PU coatings and thus the plots shown in (**c**,**d**) refer only to the PDMS-bearing coatings.

Figure 6a depicts contact angles (CA) of water, oil, and hexadecane for the PU coatings that were prepared in this study. Neat PU (prior to PDMS grafting) exhibited poor water repellency and the CA remained below 80° for water. However, the PDMS-treated PU samples showed good water repellencies with CAs reaching 110° for SCH-4. One explanation accounting for the better performance of SCH-4 could be due to the decrease in the amount of PG, which would provide sufficient NCO groups to facilitate the PDMS grafting onto its surface. In addition, the PDMS-treated PU samples (SCH-1 to 7) were subjected to abrasion tests, and a slight increase in the water CA was observed, which we believe may correspond to some minor scratches (see Figure S5).

Contact angle hysteresis (CAH) is another important parameter regarding the pinning behavior of a liquid on a surface. Figure 6b shows the water contact angle hysteresis for the PU samples, which have been significantly improved (reduced in value) after PDMS grafting. In particular, samples SCH-3 and SCH-4 showed very low hysteresis for water. These findings are also consistent with the water contact angles shown in Figure 6a. The CAs and CAH for hexadecane and vegetable oil were also determined, and are plotted in Figure 6c,d, respectively. These liquids spread on the surface of the neat PU and thus this coating did not exhibit observable contact angles or hysteresis. Meanwhile, the vegetable oil contact angles on the PDMS-treated PU showed very consistent contact angles of around 50°. In the case of hexadecane, the PDMS-treated SCH-4 coating exhibited the highest CA, which reached ~25°. The CAH of the vegetable oil varied in the range of 0.12 to 0.08 and that exhibited by the hexadecane varied in the range of 0.025 to 0.015. Among all of the samples, the PDMS-treated SCH-4 coating exhibited the lowest CAH for vegetable oil and hexadecane, which is consistent with their measured CAs.

**Figure 7:**
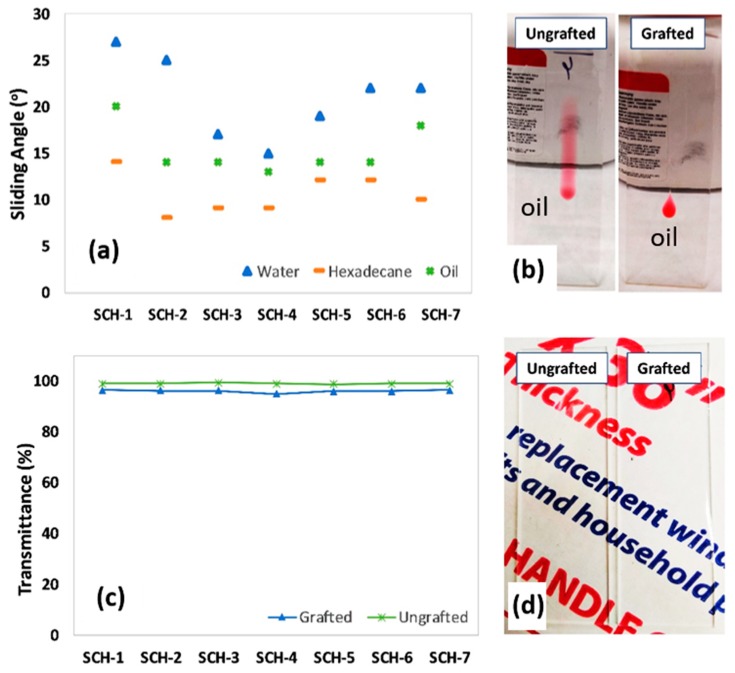
Sliding angles of water (80 µL), vegetable oil (10 µL), and hexadecane (10 µL) droplets on various PDMS-grafted PU surfaces (**a**). Oil-sliding behavior on the surfaces of PU (neat) and PDMS-PU surfaces (SCH-4) (**b**). Optical transmittance measurements revealed that the prepared coatings are quite transparent and that the grafting of PDMS did not affect the optical clarity (**c**). Photographs of PU- and PDMS-PU (SCH-4) coated glass slides (**d**).

The sliding angles of various PDMS-grafted PU samples (SCH-1 to -7) are shown in Figure 7a. Sliding angles correspond to the stickiness of a surface, and thus a low sliding angle indicates a non-sticky surface with good self-cleaning attributes. The sliding angles of water, vegetable oil, and hexadecane droplets show that oil and hexadecane spread on the PDMS-free surface, and thus these coatings lacked sliding properties. Also, the water droplets did not slide even at an inclination angle of 90°. In contrast, the PDMS-PU samples showed relatively low sliding angles against both polar and non-polar liquids. The PDMS-treated SCH-3 and SCH-4 exhibited the lowest (best) sliding angles, which was also supported by their low CAH (see Figure 6d). On the other hand, Figure 7b shows the sliding behavior of vegetable oil on the surfaces of ungrafted (neat PU) and grafted (PDMS-PU) coatings that were applied onto glass slides. The superior oil repellency of the PDMS-grafted surface relative to the neat PU is clearly evident. In fact, the neat PU allows the vegetable oil to spread on its surface.

One of the key challenges with PDMS-PU coatings arises from the macrophase separation of PDMS in a PU matrix due to the differing surface energies of PU and PDMS. Therefore, one aim of this study was to fabricate a clear coating. Consequently, the percent transmittances (%T) of all samples (SCH-1 to -7) were determined before and after PDMS grafting (Figure 7c). Prior to the PDMS grafting, the PU coating showed excellent optical clarity (99.0 ± 0.3%T), which diminished only slightly after the PDMS treatment to 96.0 ± 0.5%T. All of the samples showed fairly consistent and high %T values, which thus demonstrates that this new method can provide a useful route for the fabrication of optically clear omniphobic self-healing coatings. Figure 7d shows a photograph of glass slides that were coated with PU (without grafting) and PU with PDMS grafting. As can be seen in the picture, both of the coated slides with and without PDMS grafting exhibit excellent optical clarity. These excellent optical properties indicate the absence of macrophase separation in the prepared sample.

## 4. Conclusions

Herein we report a unique approach for the fabrication of dual-functional coatings capable of self-healing and self-cleaning. Employing PU as a bottom layer with a dynamic urethane component imparted self-healing properties, while PDMS treatment enabled self-cleaning properties. The obtained coatings showed good thermal-assisted self-healing properties. In addition, the self-cleaning behavior was somewhat dependent on the stoichiometric balance between NCO and OH (of the PG), and samples with less of PG (SCH-4) showed superior self-cleaning properties among the tested samples. Notably, all of the prepared coatings are also optically transparent, indicating the lack of phase separation of PDMS in the PU matrix. In the future, detailed microscopic analysis, as well as the characterization of the PDMS concentration gradient across the PU matrix, will be investigated. These coatings have promising potential for a variety of applications, particularly where optical clarity, liquid repellency, and self-healing performance or robustness are desired.

## Figures and Tables

**Table 1:** Formulations of the samples prepared in this study.

Sample Code	Molar Equivalent ratio (PU-prepolymer:Propyl gallate)	PU-prepolymer 75 (mg)	PG (mg)	HDIT (mg)
**SCH-1**	1.0: 1.10	2000	380	140
**SCH-2**	1.0: 1.05	2000	364	140
**SCH-3**	1.0: 1.00	2000	350	140
**SCH-4**	1.0: 0.95	2000	334	140
**SCH-5**	1.0: 0.90	2000	320	140
**SCH-6**	1.0: 0.85	2000	304	140
**SCH-7**	1.0: 0.80	2000	290	140

## Supplementary Materials

The following are available online at https://www.mdpi.com/2073-4360/11/11/1849/s1, Figure S1–S4 depict ^1^H NMR spectra of the precursors used in this study. Figure S5 illustrates the scratch resistance of the coatings, while Figure S6–S7 depict the non-healing behavior of the conventional urethane coatings.
