# Supplementary material for: Dual-Layer Approach toward Self-Healing and Self-Cleaning Polyurethane Thermosets
Source: Polymers (Basel). 2019 Nov 9;11(11):1849. doi: 10.3390/polym11111849 (PMC6918376; doi:10.3390/polym11111849)
Supplement: Supplementary file 1 [file polymers-11-01849-s001.pdf]

SUPPORTING INFORMATION

Dual-Layer Approach toward Self-Healing and Self-Cleaning Polyurethane Thermosets

Muhammad Naveed, Muhammad Rabnawaz*, Ajmir Khan, Mohammad O. Tuhin

School of Packaging, Michigan State University, 448 Wilson Road, East

Lansing, Michigan 48824-1223, United States of America

Corresponding author: Muhammad Rabnawaz

Tel.: +1 517-432-4870;

E-mail: rabnawaz@msu.edu; Postal address: School of Packaging, Michigan State University,

448 Wilson Road, East Lansing, Michigan 48824-1223, United States of America.

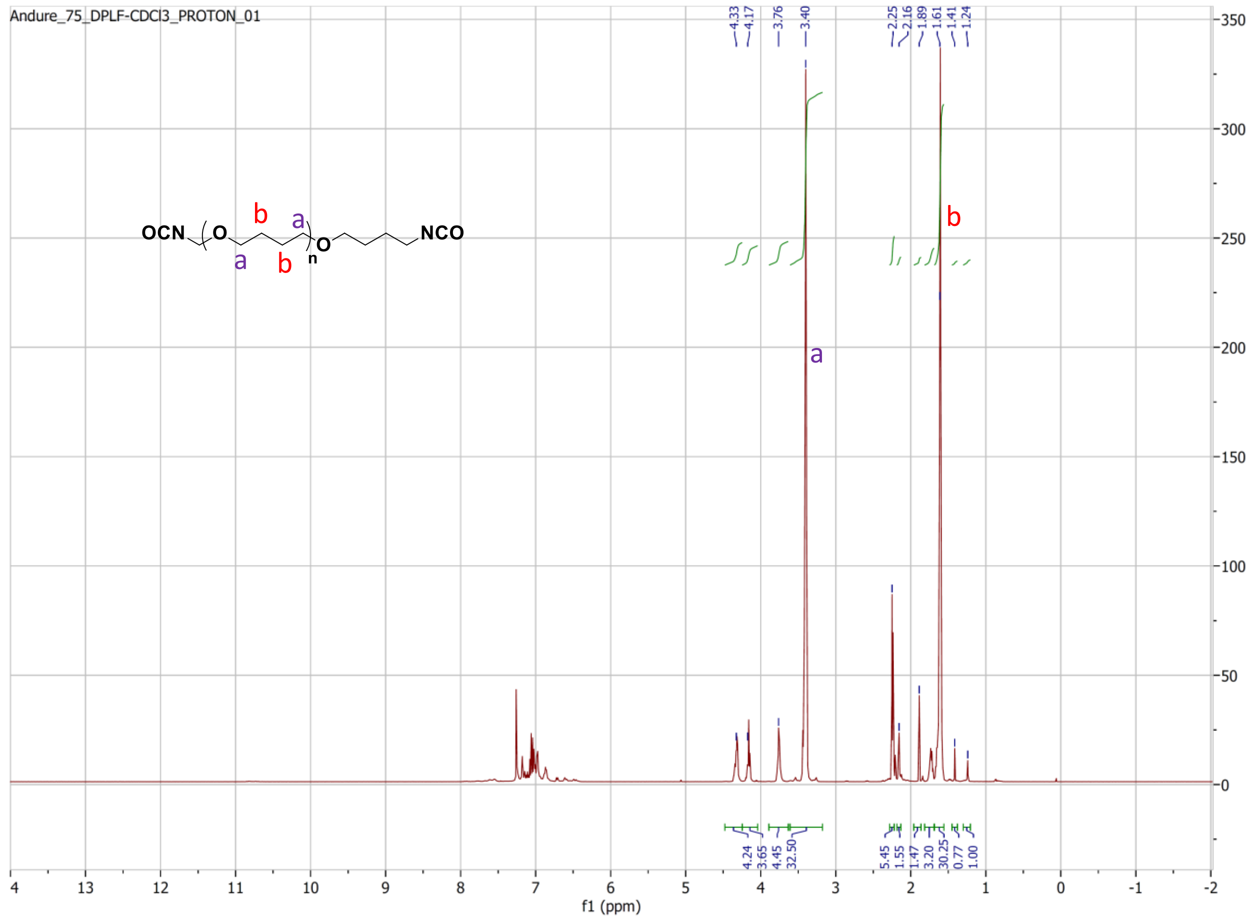

Figure S1: ^{1}H NMR (500 MHz, $CDCl_3$) of the PU pre-polymer; Strong peaks at 3.40 ppm (a) and 1.61 ppm (b) indicates polytetrahydrofuran (PolyTHF) backbone of the pre-polymer.

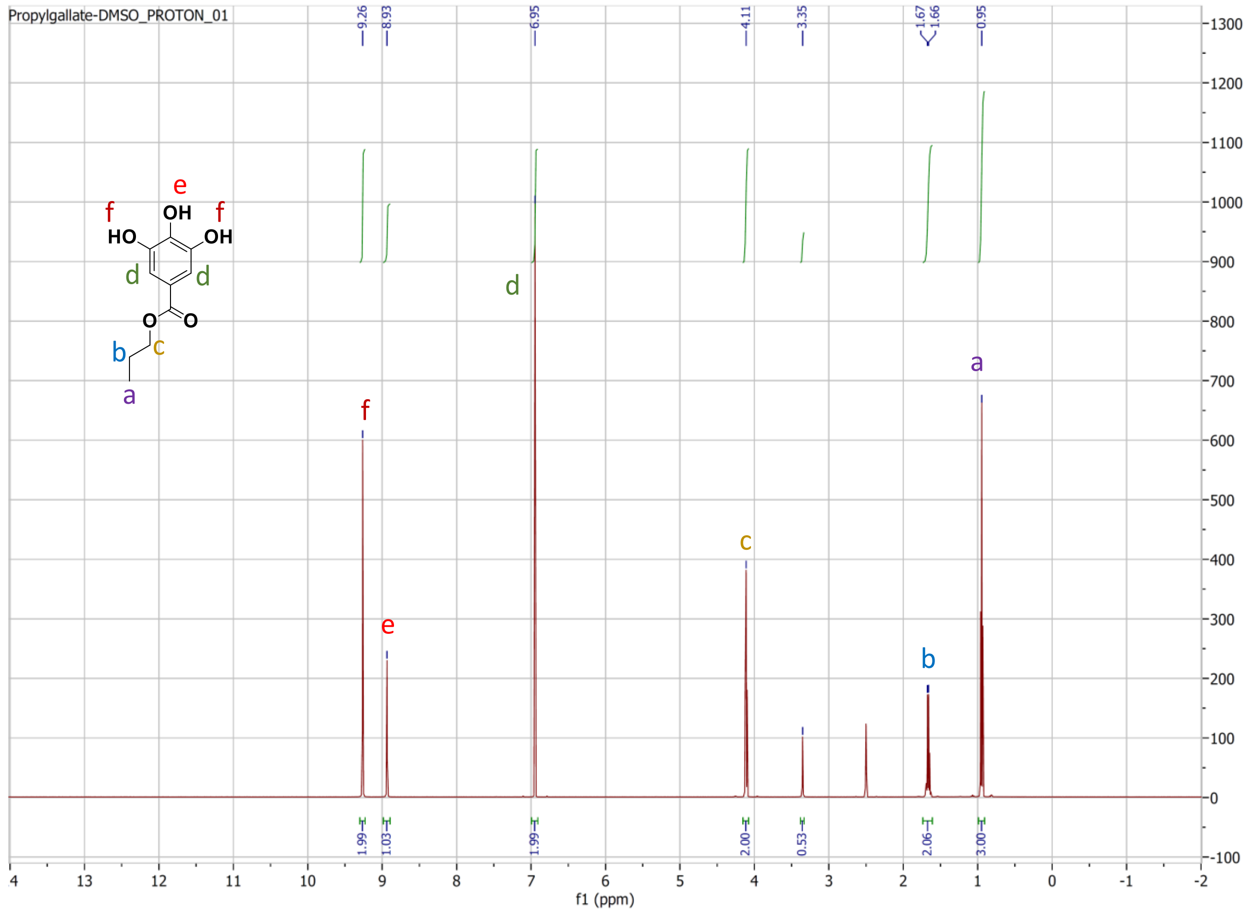

Figure S2: ^{1}H NMR (500 MHz, DMSO-d_6) of the propylgallate (PG)

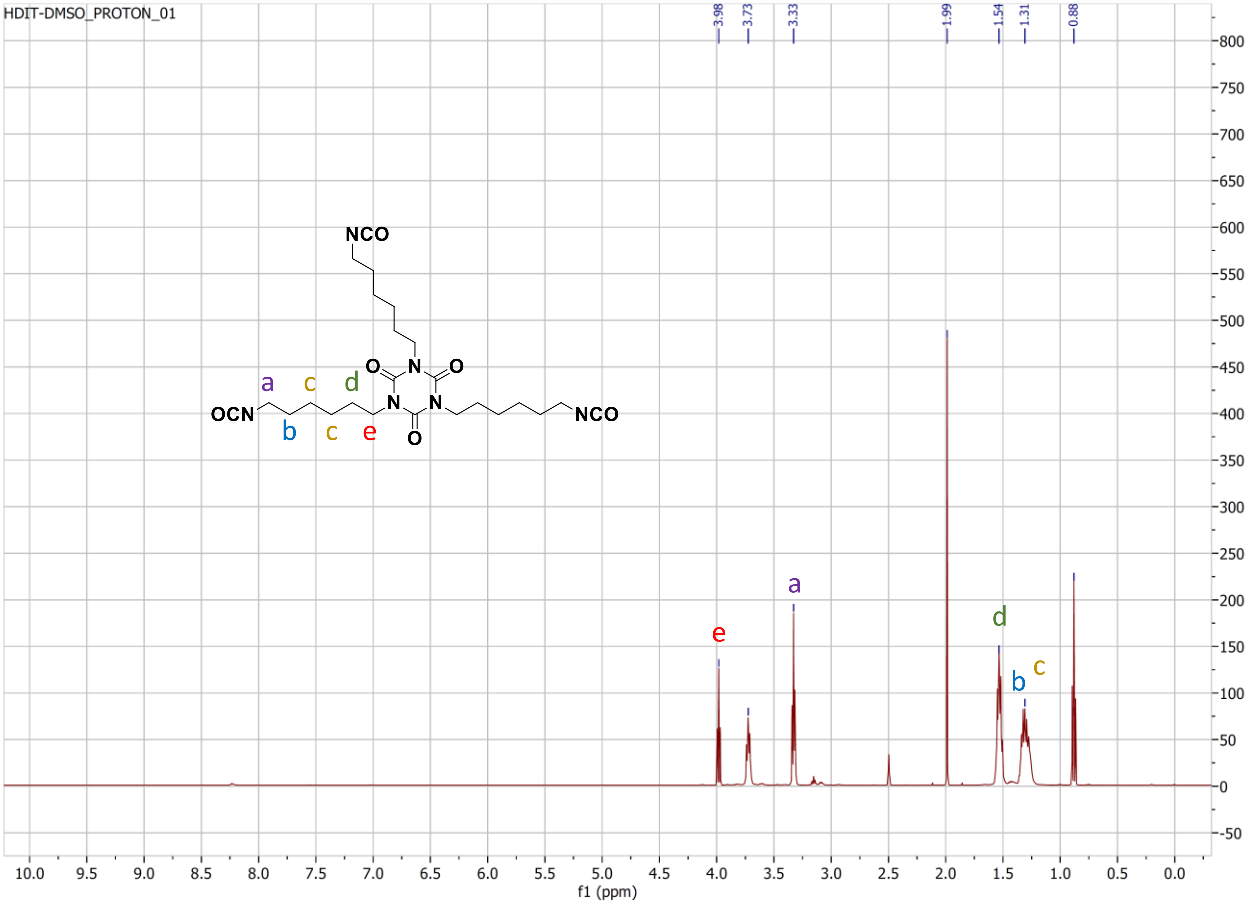

Figure S3: ^{1}H NMR (500 MHz, DMSO-d_6) of the hexamethylene diisocyanate trimer (HDIT)

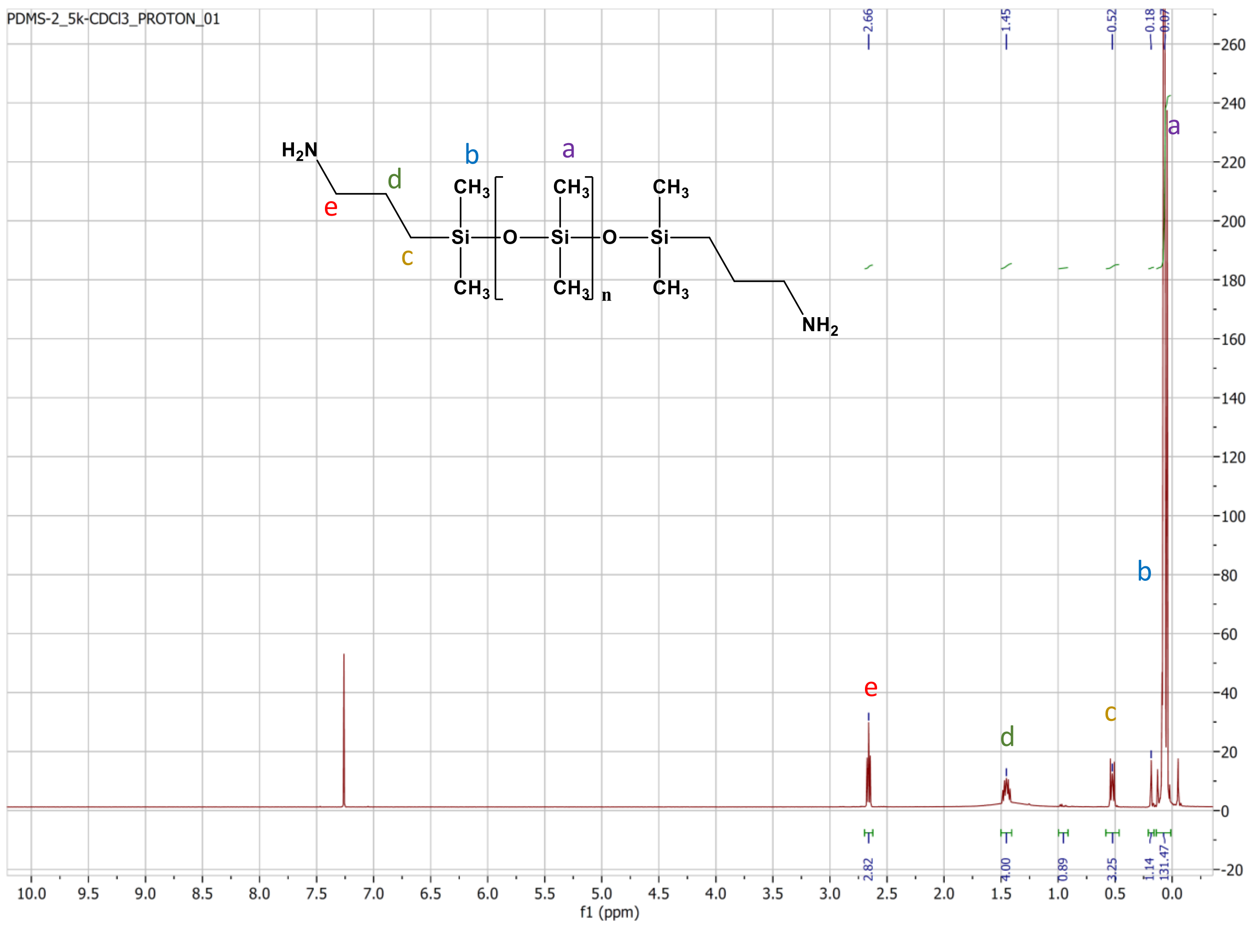

Figure S4: ^{1}H NMR (500 MHz, $CDCl_3$) of the NH_2-PDMSNH_2(M_n= 2,500 g/mol)

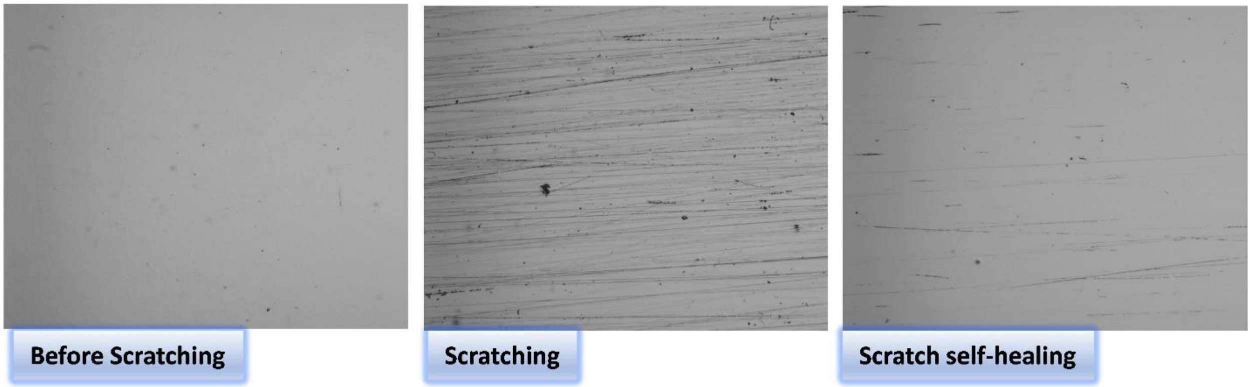

Figure S5: Optical microscopic images, scratch autonomous self-healing in two days at ambient temperature.

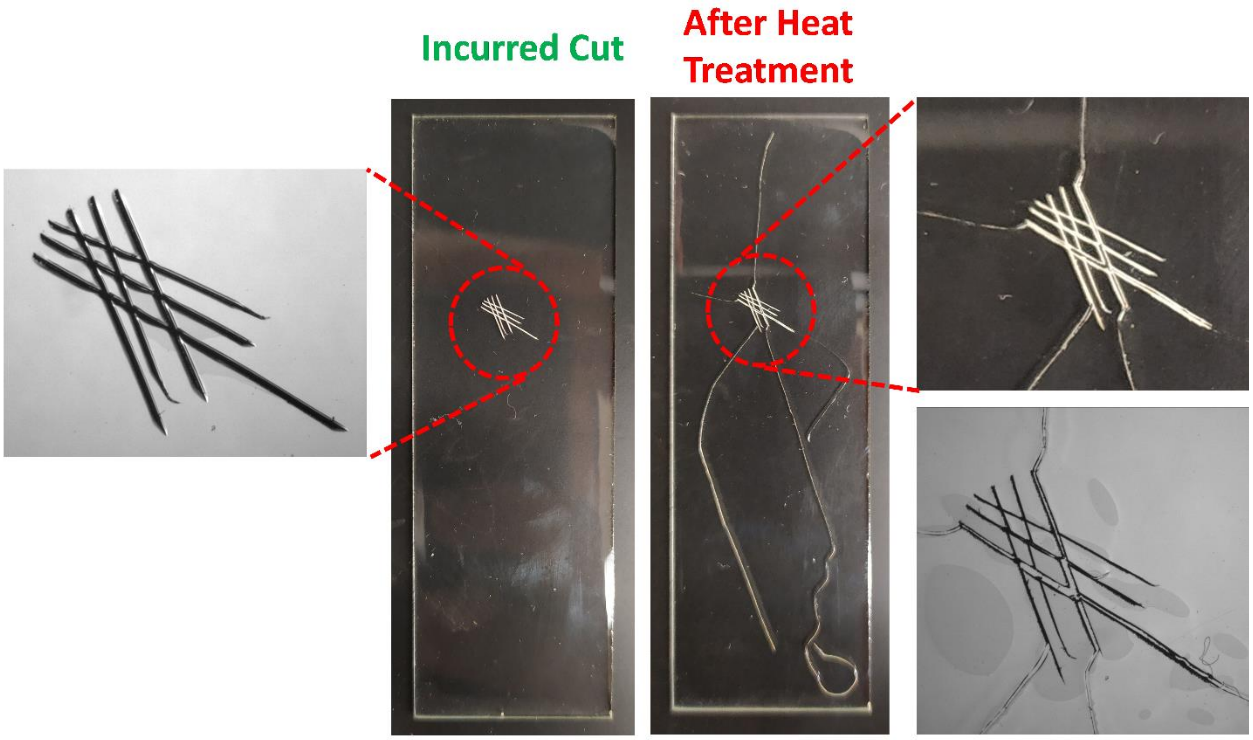

Figure S6: Digital and optical microscopy images showing the lack of self-healing ability of conventional polyurethane coating derived from hexamethylene diisocyanate trimer and acrylic polyol before and after thermal treatment at 130 ℃ for 40 min.

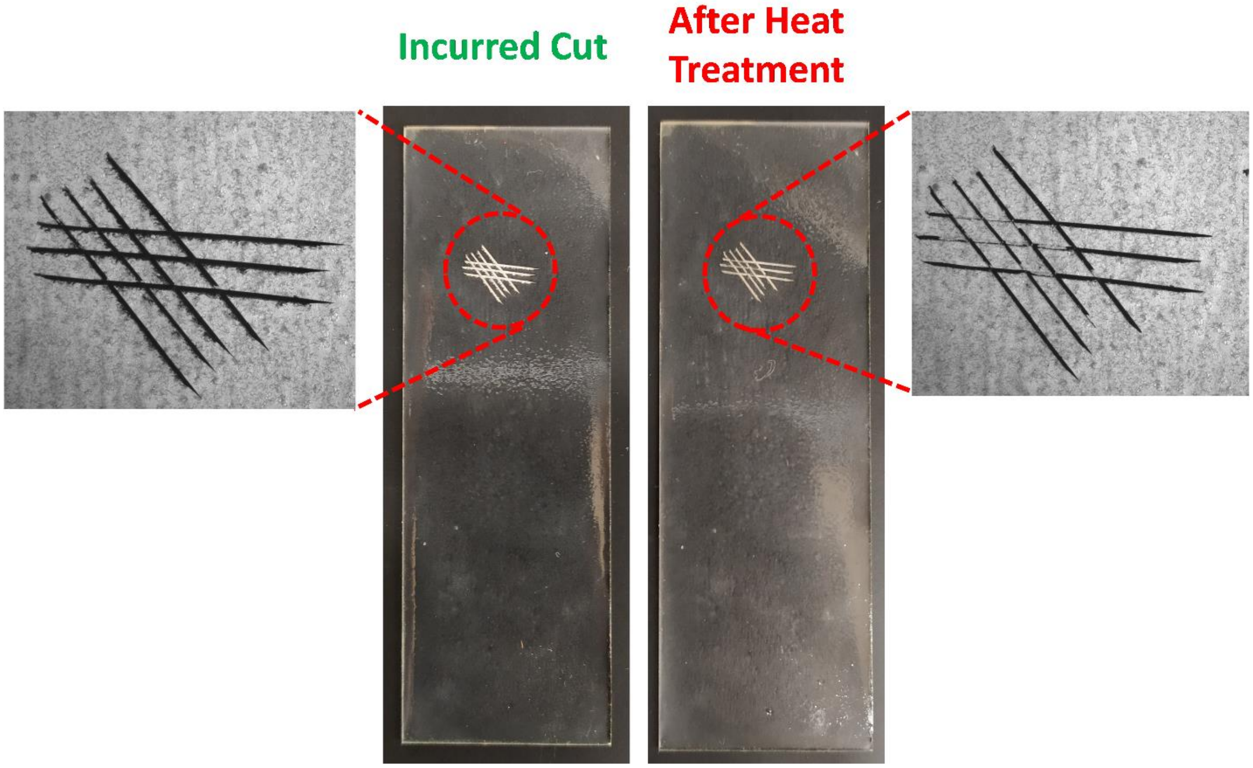

Figure S7: Digital and optical microscopy images showing the lack of self-healing ability after thermal treatment at 130 ℃ for 40 min of polyurethane coatings prepared by polyTHF PU prepolymer and glycerol.
